# Identification of Non-Invasive Diagnostic Markers for Oral Squamous Cell Carcinoma Through Salivary Microbiome and Gene Expression Analysis

**DOI:** 10.3390/ijms26168104

**Published:** 2025-08-21

**Authors:** Mitsuhiro Hishida, Kosuke Nomoto, Kengo Hashimoto, Sei Ueda, Shuji Nomoto

**Affiliations:** 1 Department of Surgery, Aichi Gakuin University, 2-11 Suemori-dori, Chikusa-ku, Nagoya 464-8651, Japan; k_npeace@yahoo.co.jp (K.N.); uedasei@mediacat.ne.jp (S.U.); snomoto@dpc.agu.ac.jp (S.N.); 2 Department of Surgery, Nagoya Central Hospital, 3-7-7 Taiko, Nakamura-ku, Nagoya 453-0801, Japan; 3 Department of Maxillofacial Surgery, Aichi Gakuin University, 2-11 Suemori-dori, Chikusa-ku, Nagoya 464-8651, Japan

**Keywords:** oral squamous cell carcinoma, saliva, salivary biomarkers, microbiome analysis, gene expression analysis

## Abstract

Oral squamous cell carcinoma (OSCC) is a malignancy with a poor prognosis, and early diagnosis is essential for improving patient survival and quality of life. This study aimed to develop a non-invasive screening method based on salivary gene expression and microbiome analysis. Unstimulated saliva samples were collected from patients with OSCC, patients with oral potentially malignant disorders, and healthy controls. Microbiome profiling was performed using 16S ribosomal RNA gene sequencing. The OSCC group showed a significant increase in *Fusobacterium* and Bacteroidetes and a decrease in *Streptococcus*. LEfSe analysis indicated microbial changes associated with disease progression. Receiver operating characteristic analysis demonstrated high diagnostic accuracy when multiple bacterial species were combined. An increase in Fusobacteria was also associated with a higher risk of recurrence. Gene expression analysis revealed that *NUS1*, *RCN1*, *CPLANE1*, and *CCL20* were significantly upregulated in OSCC, as confirmed by qRT-PCR and tissue expression data. Notably, CCL20 expression positively correlated with *Fusobacterium* abundance. These findings suggest that integrated analysis of the salivary microbiome and gene expression may offer a useful non-invasive approach for early OSCC detection and disease monitoring. Furthermore, we integrated current evidence from the literature to provide a comprehensive overview.

## 1. Introduction

Oral cancer is among the most prevalent malignancies worldwide, with more than 90% classified as oral squamous cell carcinoma (OSCC) [1], originating from the oral mucosa. Despite advancements in medical technology, the 5-year survival rate for OSCC remains stagnant at 50–60% over the past several decades [2,3]. Early detection of OSCC is crucial for improving quality of life and prognosis, highlighting the urgent need for reliable novel detection methods.

Currently, serum biomarkers for OSCC serve only as supplementary tools and are inadequate as screening modalities because they do not accurately reflect tumor status. In contrast, saliva is an easily collectible, non-invasive biofluid, more suitable for screening than blood [4,5,6,7]. In this study, we focused on the clinical applicability of oral microbiome changes associated with OSCC development.

Recent years have seen a rapid accumulation of knowledge regarding the relationship between microbiomes and cancer [8]. For instance, *Helicobacter pylori* is well known for its role in gastric cancer and MALT lymphoma [9]. Alterations in gut microbiota have been linked to numerous diseases including inflammatory bowel disease and colorectal cancer, and next-generation sequencing (NGS) has enabled detailed characterization of these microbial communities [10,11].

Although oral microbes are believed to be eliminated by gastric acid upon ingestion, recent reports have identified distinct oral microbiome profiles in patients with gastric and pancreatic cancers [12,13], suggesting the presence of disease-specific microbial patterns. Oral bacteria implicated in colorectal cancer have also been reported [14,15], supporting a strong relationship between the oral and gut microbiomes. However, the clinical utility of these microbiomes as diagnostic markers remains inconclusive.

The upper aerodigestive tract—including the oral cavity, pharynx, larynx, and esophagus—is a well-known predilection site for squamous cell carcinomas. Reports have shown that 2.2–2.7% of patients with OSCC develop synchronous or metachronous multiple primary malignancies, among which 0–30.3% involve the head and neck region [16]. Furthermore, approximately 6.7% of patients with esophageal squamous cell carcinoma have been reported to have concomitant head and neck cancers [17]. These anatomical sites are all continuously exposed to saliva—about 1.5 L produced daily—and share exposure to external factors such as mastication, swallowing, smoking, and alcohol consumption.

Leukoplakia, a premalignant lesion, is commonly observed in OSCC, particularly in mechanically stimulated areas such as the tongue and soft palate [18,19]. Esophageal leukoplakia, though rare, has also been reported, suggesting a similar role of mechanical stimulation in disease pathogenesis [20].

Previous studies have examined local microbiome changes in the tongue, hard palate, and buccal mucosa in relation to head and neck cancers [21], and recent investigations have extended to the pharynx, larynx, and esophagus. These organs, being exposed to saliva, may experience microbiome alterations influencing cancer development. Saliva provides a comprehensive reflection of the oral microbiome and, through swallowing, may impact microbial environments in downstream organs [22,23].

Saliva collection is non-invasive, simple, and less affected by circadian or dietary variation when collected in the early morning, thus offering a reliable reflection of oral conditions [24,25]. Accordingly, salivary microbiome analysis holds promise not only as a novel OSCC detection tool but also for guiding new prevention and therapeutic strategies, such as probiotic intervention.

This report integrates our findings from comparative salivary microbiome analyses of OSCC, leukoplakia, and healthy individuals [26,27], along with gene expression studies in saliva and tissue [28,29,30], to evaluate the diagnostic and prognostic potential for OSCC.

To address this, we collected saliva samples from patients with OSCC, patients with oral potentially malignant disorders (OPMDs), and healthy controls without malignancy (HC), and conducted microbiome profiling via 16S rRNA sequencing and gene expression analysis via quantitative reverse transcription polymerase chain reaction (qRT-PCR).

## 2. Results

### 2.1. Association Between Microbiome Profiles and OSCC

Using 16S rRNA gene sequencing, we comprehensively analyzed salivary microbiomes of patients with OSCC, OLK, as an OPMD, HC, and post-operative OSCC cases (Post).

Comparing the OSCC group to the non-OSCC group (OLK + HC), significant differences in microbial composition were observed. In the initial study, relative abundance analysis showed that Bacteroidetes was significantly higher and *Streptococcus* significantly lower in OSCC versus OLK (*p* < 0.05). Among genera under 1% abundance, *Solobacterium* was elevated in OSCC (*p* < 0.05), and *Porphyromonas gingivalis* increased in both OSCC and OLK but was undetected in controls. *Streptococcus anginosus* was present in all groups yet showed higher abundance in OSCC and OLK.

When including Post cases, relative abundances of *Streptococcus*, *Aggregatibacter*, and *Alloprevotella* differed significantly across OSCC, OLK, and Post groups. α diversity analysis revealed lowest diversity in Post, with OSCC and OLK similar. β diversity (weighted UniFrac) showed clear group separation. Advanced-stage OSCC tended to show higher α diversity than early-stage. Linear Discriminant Analysis Effect Size (LEfSe) analysis indicated elevated Linear Discriminant Analysis (LDA) scores for Fusobacteria, *Fusobacterium*, and Bacteroidetes in OSCC, while Firmicutes and *Streptococcus* dominated in non-OSCC. Early OSCC was enriched for *Streptococcus*, whereas advanced OSCC showed higher *Fusobacterium*, suggesting microbiome shifts with disease progression (Table 1).

The Receiver Operating Characteristic (ROC) curve analysis determined optimal relative abundance cut-offs for differentiating OSCC and non-OSCC: Fusobacteria 10.2%, *Fusobacterium* 8.5%, *Streptococcus* 11.4%, Firmicutes 25% (Bacteroidetes excluded due to low Area Under the Curve (AUC)). A multi-genus model yielded a significantly higher AUC versus single-genus models (Table 2). Among patients with recurrent or cervical lymph node metastasis within one year post-surgery (recurrence group), Fusobacteria and *Fusobacterium* had high LDA scores, while *Streptococcus* predominated in the non-recurrence group. Kaplan–Meier analysis showed that patients with Fusobacteria ≥ 13.8% (median) had significantly higher recurrence by one year.

### 2.2. Salivary Gene Expression Analysis and Its Relation to the Microbiome

qRT-PCR analysis demonstrated that salivary expression levels of nuclear undecaprenyl pyrophosphate synthase 1 (*NUS1*), a subunit of the dehydrodolichyl diphosphate synthase complex involved in dolichol biosynthesis and N-linked protein glycosylation [31], and Reticulocalbin 1 (*RCN1*), an endoplasmic reticulum resident calcium-binding protein implicated in endoplasmic reticulum calcium homeostasis and the regulation of cell proliferation [32], were significantly elevated in OSCC patients compared with healthy controls. Their combined use yielded a sensitivity of 0.927 and specificity of 0.707.

Ciliogenesis and Planar Polarity Effector 1 (*CPLANE1*), associated with autosomal recessive ciliopathic OFD6/Varadi syndrome [33], was significantly overexpressed in the OSCC group compared to HC and OLK (*p* < 0.001). Its expression correlated with differentiation grade (*p* = 0.002), but not with tumor size, stage, or nodal metastasis. ROC analysis revealed an AUC of 0.908, sensitivity of 0.814, and specificity of 0.925 for CPLANE1, but no correlation with *Fusobacterium* abundance (Table 3).

C-C Motif Chemokine Ligand 20 (*CCL20*), a key mediator in the tumor microenvironment [34], was also significantly upregulated in OSCC saliva, with even higher levels in advanced-stage cases (*p* = 0.004). NGS and LEfSe analyses identified *Fusobacterium*, *Porphyromonas*, and *Treponema* as characteristic of OSCC. Notably, *Fusobacterium* abundance correlated positively with CCL20 expression (Spearman ρ = 0.426, *p* = 0.019) (Table 4). The ROC analysis for CCL20 (cut-off 0.069) showed a specificity of 0.983 and positive predictive value (PPV) of 0.979.

Comparing OSCC patients to OPMD + HC, CCL20 had a specificity of 0.983 and a PPV of 0.979. Against HC alone, the specificity was 0.980 and the PPV was 0.979, with a high sensitivity and negative predictive value (NPV). The diagnostic performance of these salivary biomarkers is summarized in Table 3.

## 3. Discussion

In this study, we proposed a novel approach to the early diagnosis and elucidation of the pathogenesis of OSCC by characterizing the salivary microbiome and gene expression profiles associated with OSCC and its precursor lesions—OPMDs, including OLK.

First, analysis of the salivary microbiome revealed significant differences in microbial composition among the disease groups. Notably, in OSCC patients, the relative abundances of specific bacterial genera such as *Solobacterium*, *Fusobacterium*, *Porphyromonas gingivalis*, and *Streptococcus anginosus* were increased, suggesting that these taxa may be involved in carcinogenesis and tumor progression. Conversely, certain members of the *Streptococcus* genus were decreased in OSCC, indicating that the microbial dysbiosis in OSCC involves not only the presence or absence of specific bacteria but also a complex shift in overall bacterial balance. Other studies have also supported a strong association between salivary microbiome composition and OSCC development [35,36,37,38].

Traditionally, risk factors for OSCC have included smoking, alcohol consumption, and viral infections; however, recent evidence indicates that these factors cannot fully explain all cases. There is a growing recognition that poor oral hygiene and abnormalities in the oral microbiome may serve as novel risk factors for OSCC [39,40]. In fact, oral commensal bacteria have been detected in cervical lymph node metastases and tumor tissues from OSCC patients [41], supporting the hypothesis that these bacteria may contribute to tumor initiation and progression.

Additionally, salivary samples from OSCC patients exhibited trends in microbial diversity and composition that varied with disease progression. This suggests that tumor growth may alter the tissue environment, creating conditions favorable for colonization by specific bacteria [42]. Furthermore, patients with a higher abundance of Fusobacteria exhibited significantly increased recurrence rates within one year post-surgery, implying that specific microbial profiles may be useful for prognostic prediction.

The maintenance of immune homeostasis critically depends on healthy microbiota, which also plays a pivotal role in tumor immune surveillance. Conversely, dysbiosis, characterized by reduced microbial diversity and an overgrowth of pathogenic bacteria in the gut, has been implicated in cancer initiation and progression. Such microbial imbalances may promote tumorigenesis through mechanisms involving chronic inflammation, metabolic disturbances, and impaired immune regulation [43,44,45]. Several hypotheses explain the role of specific pathogens in shaping the tumor microenvironment. The “alpha-bug hypothesis” posits that a single pathogenic species can reshape the microbial community, creating a tumor-promoting niche. The “driver–passenger model” suggests that initial colonizers (drivers) initiate inflammation and DNA damage, followed by the accumulation of adapted bacteria (passengers), which further contribute to tumor development. Furthermore, certain bacterial genotoxins, such as those produced by *enterotoxigenic Bacteroides fragilis* (ETBF), *Escherichia coli*, and *Salmonella*, have been shown to induce DNA damage, thereby promoting tumorigenesis. Chronic inflammation mediated by specific microbes may stimulate cytokine production and signal transduction pathways, leading to tumor proliferation and immune suppression. Commensal gut microbes also influence T cell differentiation, exhaustion, and apoptosis, while B cells contribute to immune suppression. Moreover, dysbiosis-induced recruitment of immunosuppressive cells may facilitate metastasis by promoting tumor colonization at distant sites [45].

Next, mRNA expression analysis of salivary samples revealed the expression dynamics of several gene markers (NUS1, RCN1, CPLANE1, and CCL20), and we examined their diagnostic potential and association with disease characteristics. NUS1 and RCN1 were selected based on their significantly higher expression in OSCC tissues (more than twofold compared to adjacent non-cancerous tissue), supported by previous reports of their roles in other cancers. CPLANE1 expression was notably elevated in OSCC compared to both healthy volunteers and patients with OPMDs, demonstrating its specificity for malignant transformation. CCL20 was selected based on previous studies demonstrating its increased expression (approximately 1.8-fold) in OSCC samples and its association with *Fusobacterium* colonization in esophageal cancer.

Gene expression differences were further validated by expanding comparisons beyond OSCC and healthy controls to include OPMDs and oral microbiome profiles. Although gene expression analysis in OPMD tissues was limited by the small sample size available from diagnostic biopsies, we employed the Gene Expression database of Normal and Tumor tissues 2 (GENT2) to objectively evaluate tissue expression levels for CPLANE1 and CCL20.

In all analyses, the expression of these genes differed significantly between OSCC patients and HCs, highlighting saliva’s promise as a non-invasive source for biomarker detection.

Although NUS1 and RCN1 showed high expression in tissue microarrays, no significant differences were observed between tumors and adjacent tissues by qRT-PCR, whereas clear differences were detected in saliva. This may reflect tumor-associated environmental factors rather than direct expression by tumor cells. Given their involvement in different oncogenic pathways, the combined use of these genes as multi-markers may enhance diagnostic accuracy. In contrast, CPLANE1 expression increased progressively from healthy controls to OPMDs and OSCC patients, correlating with histological differentiation, indicating its potential as an early carcinogenesis marker. Its high diagnostic performance (sensitivity 0.814, specificity 0.925) and elevated expression in OPMDs also suggest utility in screening for precancerous lesions. CCL20 was consistently upregulated in both OSCC tissues and saliva, particularly in advanced stages, implying a role in tumor progression. These markers reflect distinct stages and aspects of OSCC, and their combined application may improve staging accuracy, reduce false positives, and contribute to the development of more precise screening strategies.

The utility of salivary transcriptomics for the non-invasive diagnosis and prognostic prediction of OSCC has also been supported by other studies [46,47].

Recent advancements have explored NGS-based proteomic approaches to identify epithelial cell-derived biomarkers in saliva [48], as well as integrated analyses using public databases such as The Cancer Genome Atlas (TCGA) and Gene Expression Omnibus (GEO) to assess gene expression profiles and survival outcomes in OSCC, particularly among non-smokers [49,50,51].

Furthermore, our study identified associations between gene expression and the salivary microbiome. Recent clinical studies have reported links between *Fusobacterium* species in the oral cavity and cancer development and progression [52,53,54]. In our current analysis, no correlation was found between salivary CPLANE1 expression and the abundance of oral bacteria, including *Fusobacterium*. In contrast, a positive correlation was observed between CCL20 expression in saliva and *Fusobacterium* abundance in the OSCC group, suggesting a possible association. While elevated expression of CCL20 has also been reported to be associated with cancer in other studies [55,56], the observed correlation between the commensal bacterium *Fusobacterium nucleatum* and the chemokine CCL20 has been documented to support the involvement of the microbiome–immune axis in the pathogenesis of OSCC [57,58].

Such interactions between gene expression and the microbiome in OSCC have also been implicated in tumor progression in recent studies [59,60].

Recent advances in microbiome research have further highlighted the potential connections between the oral and gut ecosystems [61]. For instance, studies on patients with inflammatory bowel disease (IBD) have revealed that their gut microbiota composition resembles that of the oral microbiota, suggesting the translocation of oral bacteria into the gut. Possible mechanisms for this include decreased gastric acidity, altered bile acid composition, compromised intestinal barrier function, and hematogenous dissemination from damaged oral tissues [62,63].

Moreover, several studies have reported that the oral microbiota may contribute to the development and progression of colorectal cancer (CRC) [14]. Specific oral bacteria, including *Peptostreptococcus stomatis*, *Streptococcus anginosus*, *Streptococcus koreensis*, and *Solobacterium moorei*, have been implicated in CRC carcinogenesis, with *S. moorei* also potentially involved in CRC progression [15].

Our laboratory’s research also suggests that *Streptococcus* anginosus and *Solobacterium* species may be associated with OSCC. These findings underscore the importance of elucidating how the oral microbiota influences both gut microbiota composition and carcinogenic processes. Such insights will be crucial for the development of future strategies for disease prevention, diagnosis, and therapy.

## 4. Materials and Methods

### 4.1. Study Subjects and Sample Collection

In this study, unstimulated saliva samples were collected from 48 patients with OSCC, 37 with OPMDs, 20 post-operative OSCC cases, and 50 healthy controls at the Aichi Gakuin University Dental Hospital and affiliated institutions (Table 5). All unstimulated saliva samples were collected using commercial saliva collection kits specifically designed to preserve both DNA and RNA (DNA Genotek Inc., Ottawa, ON, Canada).

The study protocol was approved by the ethics committees of Aichi Gakuin University School of Dentistry and its affiliated hospitals. All procedures were conducted in accordance with the Declaration of Helsinki and the Good Clinical Practice guidelines. Written informed consent was obtained from all participants prior to enrollment.

### 4.2. DNA and RNA Extraction from Saliva

Microbial DNA was extracted from saliva using the Oragene^®^ DNA self-collection kit (DNA Genotek Inc., Ottawa, ON, Canada) according to the manufacturer’s protocol. For mRNA extraction, the Oragene^®^ RNA kit (DNA Genotek Inc., Ottawa, ON, Canada) was used, and the extracted mRNA was reverse transcribed into cDNA using the QuantiTect Reverse Transcription Kit (Qiagen, Hilden, Germany).

### 4.3. Oral Microbiome Analysis

To analyze the oral microbiota, 16S rRNA gene sequencing was performed using saliva-derived microbial DNA. Alpha and beta diversity analyses were conducted using QIIME software scripts (version 1.9.1) to assess microbial diversity across sample groups. To identify taxa with significant group-specific differences, LEfSe was used, and taxa with LDA scores greater than 4.0 were considered candidate OSCC-associated bacteria.

### 4.4. Salivary mRNA Expression Analysis

Initial exploratory gene expression profiling was performed using microarray analysis on saliva samples from four OSCC patients and four healthy controls. Genes that were markedly upregulated in OSCC samples compared to controls were selected for further evaluation. Additional candidate genes were chosen based on prior reports of their involvement in other cancer types or their putative relevance to oral tissue function.

qRT-PCR was then performed on saliva samples collected from individual participants to measure the expression levels of the selected genes. For tissue-based gene expression comparisons, qRT-PCR was performed using tissue specimens from the same individuals, and/or analysis was conducted using GENT2 to evaluate expression patterns in normal versus tumor tissues (http://gent2.appex.kr/gent2/, accessed on 28 September 2020).

### 4.5. Statistical Analysis

All statistical analyses were performed using R software (version 3.4.0, The R Foundation for Statistical Computing, Vienna, Austria). The Mann–Whitney U test, Fisher’s exact test, and chi-squared test were used for univariate analysis and comparison of relative abundance. Multivariate analysis was conducted using logistic regression. LEfSe analysis was performed using the Galaxy/Huttenhower Lab platform (https://huttenhower.sph.harvard.edu, accessed on 17 October 2018). A *p*-value of <0.05 was considered statistically significant.

Based on these methods, we investigated the relationships among OSCC, the oral microbiome, expression levels of candidate diagnostic genes, and their potential correlations.

## 5. Conclusions

Advances in salivary microbiome and gene expression profiling have opened new avenues for understanding the pathogenesis and improving the diagnosis of OSCC and its precursor lesions. Notably, alterations in bacterial composition—such as an increase in *Fusobacterium*—are implicated in tumor initiation, disease progression, and unfavorable prognosis. Salivary gene expression markers reflect various pathological states and disease stages, and their combined use may enhance diagnostic accuracy.

Furthermore, the observed correlation between *Fusobacterium* abundance and CCL20 expression suggests that microbial modulation of tumor-associated gene expression plays a role in OSCC development. This highlights the potential of integrative microbiome–transcriptome analyses in improving diagnostic precision.

Given saliva’s non-invasiveness and ease of collection, assays targeting these biomarkers and microbiome profiles have a high translational potential.

## Figures and Tables

**Table 1:** LEfSe analysis results of salivary samples in early-stage and advanced-stage OSCC.

Rank	Early-Stage OSCC	Advanced-Stage OSCC
1	g *Streptococcus*	g *Fusobacterium*
2	N.S.	g *Alloprevotella*
3	N.S.	g *Catonella*

N.S.: not significant.

**Table 2:** Results of ROC curve analysis for each bacterium in OSCC and non-OSCC groups.

Candidate Bacteria	Sensitivity/Specificity	AUC	Cut-Off Value (%)
p Fusobacteria	0.689/0.732	0.703	≥10.2
g *Fusobacterium*	0.689/0.732	0.722	≥8.50
p Firmicutes	0.756/0.659	0.745	<25
g *Streptococcus*	0.578/0.928	0.831	<11.4
Combination of four candidate bacteria	0.512/0.844	0.836 (good)	―
* p Bacteroidetes	0.829/0.533	0.632 (poor)	≥25.8

*: p Bacteroidetes was excluded due to its low AUC, and the remaining four taxa were evaluated in combination. OSCC: oral squamous cell carcinoma; ROC: Receiver Operating Characteristic; AUC: Area Under the Curve.

**Table 3:** Diagnostic accuracy of salivary biomarkers for OSCC (OSCC vs. Non-OSCC).

Biomarker	Cut-Off Value	AUC	Sensitivity	Specificity	Positive Predictive Value	Negative Predictive Value
NUS1	0.005	0.715 (0.567–0.862)	0.683 (0.519–0.819)	0.700 (0.348–0.933)	0.903 (0.742–0.980)	0.350 (0.154–0.592)
RCN1	0.041	0.759 (0.628–0.889)	0.683 (0.519–0.819)	0.900 (0.555–0.997)	0.966 (0.822–0.999)	0.409 (0.207–0.636)
NUS1 + RCN1	―	―	0.927 (0.801–0.985)	0.700 (0.348–0.933)	0.927 (0.801–0.985)	0.700 (0.348–0.933)
CPLANE1	0.001	0.908 (0.832–0.968)	0.814 (0.666–0.916)	0.925 (0.844–0.972)	0.854 (0.708–0.944)	0.902 (0.817–0.957)
CCL20	0.069	0.829 (0.839–0.946)	0.635 (0.515–0.744)	0.983 (0.909–1.000)	0.979 (0.889–0.999)	0.682 (0.572–0.779)

OSCC: oral squamous cell carcinoma; AUC: Area Under the Curve; NUS1: nuclear undecaprenyl pyrophosphate synthase 1 homolog; RCN1: Reticulocalbin 1; CPLANE1: Ciliogenesis and Planar Polarity Effector 1; CCL20: C-C Motif Chemokine Ligand 20. ( ): 95% CI.

**Table 4:** Association between OSCC, *Fusobacterium*, and gene expression.

Category	Item	Result	*p*-Value
*Fusobacterium* Abundance Comparison	OSCC vs. non-OSCC	Significantly higher in OSCC group	*p* = 0.001
	Early-stage vs. Advanced-stage OSCC	No significant difference	*p* = 0.198
Correlation Between *Fusobacterium* and Gene Expression in the OSCC Group	*Fusobacterium* vs. CPLANE1 (OSCC group)	No significant difference	*p* = 0.203
	*Fusobacterium* vs. CCL20 (OSCC group)	Significant positive correlation	*p* = 0.019

OSCC: oral squamous cell carcinoma; CPLANE1: Ciliogenesis and Planar Polarity Effector 1; CCL20: C-C Motif Chemokine Ligand 20.

**Table 5:** Characteristics of the study participants.

		OSCC (%)	OPMDs (%) (Including OLK)	Post (%)	HC (%)
Number		48	37	20	50
Mean age, years [range]		68.9 [28–92]	65.8 [29–91]	68.2 [29–85]	57.5 [26–91]
Gender	Male	35 (73)	20 (54)	14 (70)	27 (54)
	Female	13 (27)	17(46)	6 (30)	23 (46)

OSCC: oral squamous cell carcinoma; OPMDs: oral potentially malignant disorders; OLK: oral leukoplakia; Post: post-operative OSCC cases; HC: healthy controls.

## Data Availability

The datasets generated and/or analyzed during the current study are not publicly available due to ethical restrictions and participant privacy considerations, but are available from the corresponding author on reasonable request.

## Abbreviations

The following abbreviations are used in this manuscript:

OSCC	oral squamous cell carcinoma
OPMDs	oral potentially malignant disorders
Post	post-operative OSCC cases
HC	healthy controls
OLK	oral leukoplakia
IBD	inflammatory bowel disease
CRC	colorectal cancer
NGS	next-generation sequencing
LEfSe	Linear Discriminant Analysis Effect Size
LDA	Linear Discriminant Analysis
ROC	Receiver Operating Characteristic
AUC	Area Under the Curve
qRT-PCR	quantitative reverse transcription polymerase chain reaction
PPV	positive predictive value
NPV	negative predictive value
GENT2	Gene Expression database of Normal and Tumor tissues 2
TCGA	The Cancer Genome Atlas
GEO	Gene Expression Omnibus
DEGs	differentially expressed genes
NUS1	nuclear undecaprenyl pyrophosphate synthase 1 homolog
RCN1	Reticulocalbin 1
CPLANE1	Ciliogenesis and Planar Polarity Effector 1
CCL20	C-C Motif Chemokine Ligand 20
